# Supplementary material for: A contracting Intertropical Convergence Zone during the Early Heinrich Stadial 1
Source: Nat Commun. 2023 Aug 4;14:4695. doi: 10.1038/s41467-023-40377-9 (PMC10403598; doi:10.1038/s41467-023-40377-9)
Supplement: Supplementary file 1 — Supplementary information [file 41467_2023_40377_MOESM1_ESM.pdf]

Supplementary Information for

A contracting Intertropical Convergence Zone during the Early Heinrich Stadial 1

Yiping Yang[1], Lanlan Zhang[1*], Liang Yi[2], Fuchang Zhong[1], Zhengyao Lu[3], Sui Wan[1], Yan Du[4,5], Rong Xiang[1*]

[1]Key Laboratory of Ocean and Marginal Sea Geology, South China Sea Institute of Oceanology, Chinese Academy of Science, Guangzhou 510301, China

[2] State Key Laboratory of Marine Geology, Tongji University, Shanghai 200092, China

[3] Department of Physical Geography and Ecosystem Science, Lund University, Lund 22362, Sweden

[4] State Key Laboratory of Tropical Oceanography, South China Sea Institute of Oceanology, Chinese Academy of Science, Guangzhou 510301, China

[5] University of Chinese Academy of Sciences, Beijing 100049, China

Corresponding emails: llzhang@scsio.ac.cn; rxiang@scsio.ac.cn

This PDF file includes:

1. Oceanographic conditions

2. Relationship between modern $\delta^{18}O_{seawater}$ and sea surface salinity (SSS) values

3. The validity test of the Mg/Ca-temperature reconstruction

4. Supplementary Fig. 4

5. Supplementary Table 1

6. Supplementary Table 2

7. Supplementary References

1．Oceanographic conditions

Core I106 was extracted from the Ninety East Ridge in the southernmost Bay of Bengal (BoB) during a scientific cruise by the vessel the Shiyan 3. There is a low-salinity surface "boundary layer" in the BoB during the entire year, resulting from river runoff and direct precipitation over the ocean[1]. In the study area, sea surface temperature (SST) and sea surface salinity (SSS) remain relatively constant throughout the year (*i.e.*, at ~28.2-29.7°C and 33-34 psu) (Supplementary Fig.1a). Precipitation occurs year-round, with more than 69% precipitation occurring in the latter half of the year (May-December) (Supplementary Fig.1b). The changing trend in SSS is opposite to that of precipitation in study area (Supplementary Fig.1b). The lowest SSS occurs from August to November due to the highest precipitation. Therefore, monthly mean values on wind speed, SSS and precipitation over a long-time scale suggest that SSS changes in study area are closely related to precipitation.

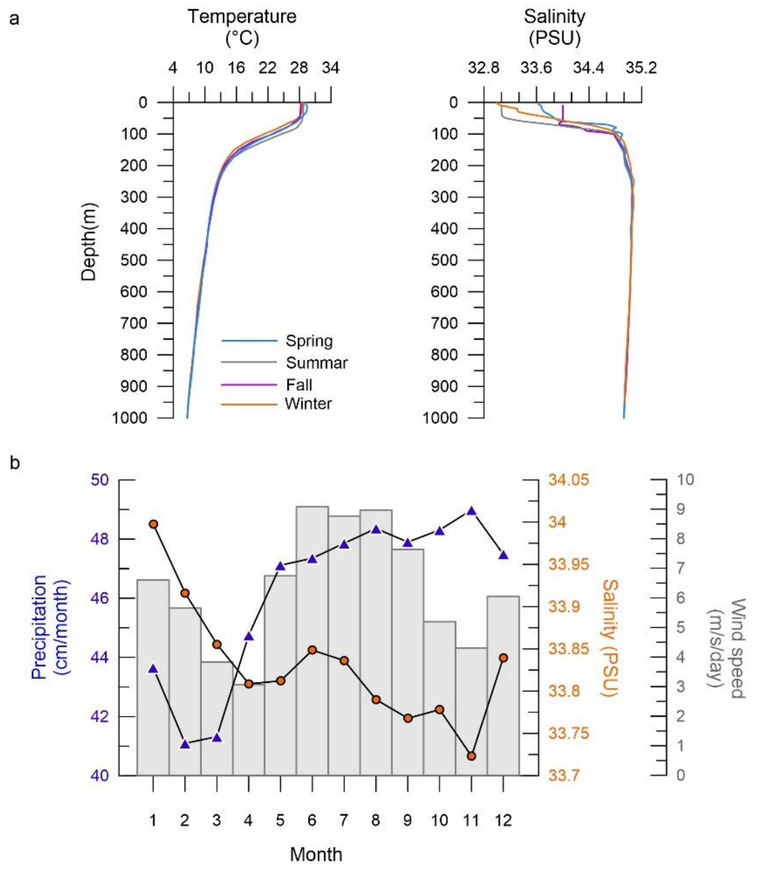

Supplementary Fig.1 Modern hydrological data from the study area. (a) Modern depth profiles of temperature and salinity in the study area, taken from the World Ocean Atlas 2018 (www.ncei.noaa.gov); (b) Mean monthly precipitation, mean monthly sea surface salinity (SSS), mean monthly wind speeds (1948-2022, http://apdrc.soest.hawaii.edu/las/v6/dataset?catitem=16341) in the study area.

2. Relationship between modern $\delta^{18}O_{seawater}$ ($\delta^{18}O_{sw}$) and SSS values

Modern observed data from the Andaman Sea[1] and the southern BoB[2,3,4] all suggest that there is a linear correlation between $\delta^{18}O_{sw}$ and salinity (Supplementary Fig.2). In study area, the quantity of freshwater in sea surface and SSS is ultimately controlled by the monsoonal precipitation that has fallen in preceding years[1]. The $\delta^{18}O$ values measured from several *G. ruber* shells in this study can be taken as mean multiyear signals. The reconstructed changes in $\delta^{18}O_{sw}$ can therefore be principally associated with the variable quantities of surficial freshwater[1]. Multiple studies of the eastern Indian Ocean have used $\delta^{18}O_{sw}$ records reconstructed from Mg/Ca ratios and *G. ruber* shell $\delta^{18}O$ datasets as a proxy of salinity to decipher the variability in Indian Ocean Summer Monsoon (IOM) precipitation levels[5,6,7].

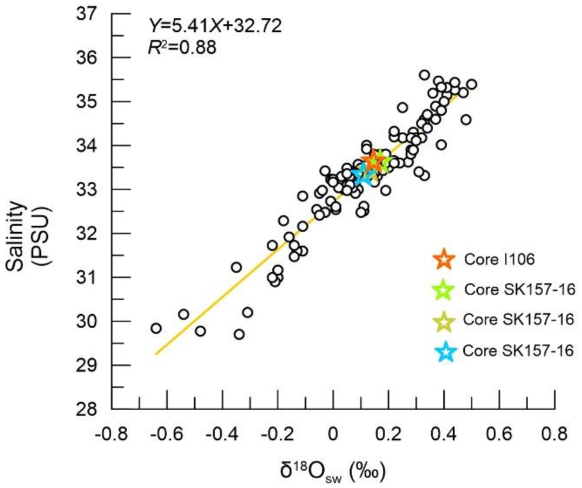

Supplementary Fig.2 Modern $\delta^{18}O_{seawater}$ ($\delta^{18}O_{sw}$) and salinity data from the Andaman Sea[1], and the southern BOB[2,3,4] along with reconstructed $\delta^{18}O_{sw}$ values calculated from *G. ruber* $\delta^{18}O$ values for the Late Holocene (2-0 ka BP), and modern sea surface temperatures (SSTs), for four core locations along Ninety East Ridge in the southern BoB[1,8]. Reconstructed $\delta^{18}O_{sw}$ values for the four cores were calculated using the $\delta^{18}O$-temperature calibration equation proposed by Bemis et al. (1998) [9].

3. The validity test of the Mg/Ca-temperature reconstruction

In order to assess the validity of the Mg/Ca-temperature reconstruction in core I106 and other records in this study, we utilized the Paleo-Seawater Uncertainty Solver (PSU Solver) to evaluate the impact of salinity on Mg/Ca. PSU Solver is a computational toolkit which calculates Mg/Ca-temperature and $\delta^{18}O_{sw}$ using an iterative approach with many uncertainty constraints[10]. PSU can test the robustness of past climate variability developed from paired Mg/Ca and $\delta^{18}O$ measurements. We evaluate the effect of salinity on Mg/Ca variability of cores I106, ADM-159[11], MD97-2141[12] and SK129/CR04[13] by PSU Solver with two signa analytical uncertainty of Mg/Ca and $\delta^{18}O$ and

±5% age uncertainty. The recalculated Mg/Ca-temperature results by PSU Solver in cores I106, ADM-159, MD97-2141 and SK129/CR04 are consistent with their published data in these studies (Supplementary Fig.3). The potential effects of salinity on these temperature results are very minor in these studies.

Moreover, we also recalculated $\delta^{18}O_{sw}$ results by PSU Solver without correcting for the effect of ice volume for cores ADM-159, MD97-2141 and SK129/CR04. $\delta^{18}O_{sw}$ values in core SK129/CR04 were recalculated by equation of Bemis et al.[9] in the PSU, as there was not $\delta^{18}O_{sw}$ equation of Epstein et al.[14] as original paper in PSU Solver. Overall, the changing trends of $\delta^{18}O_{sw}$ values in cores I106, ADM-159, MD97-2141 and SK129/CR04 are also consistent with their original dates. And their $\delta^{18}O_{sw}$ results reconstructed by PSU Solver all exist a negative abnormal event during the early Heinrich stadial 1 (HS1). Therefore, we believe that these Mg/Ca and $\delta^{18}O_{sw}$ values in these cores can still robustly reconstruct the paleo-climate.

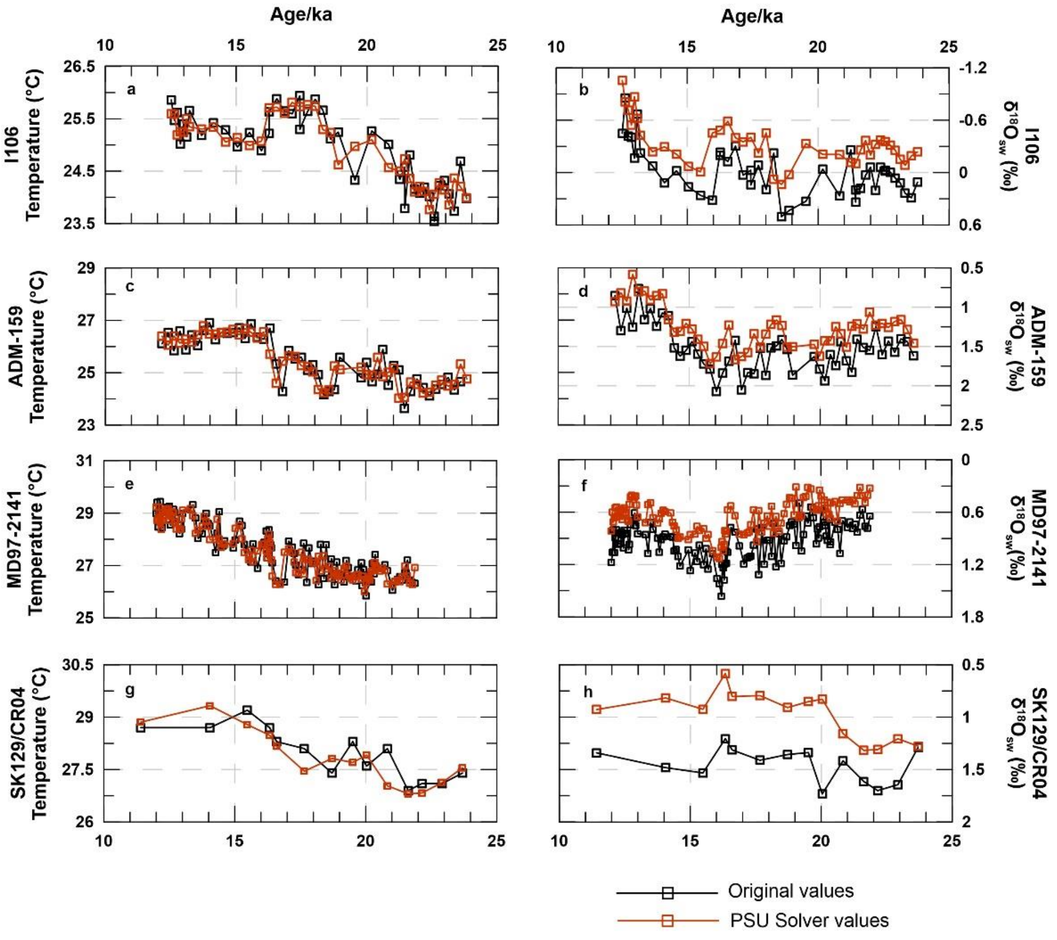

Supplementary Fig.3 Comparison of original and Paleo-Seawater Uncertainty Solver (PSU Solver) of Mg/Ca-temperature (a, c, e, g) and $\delta^{18}O_{seawater}$ ($\delta^{18}O_{sw}$) (b, d, f, h) values in cores I106, ADM-159[11], MD97-2141[12] and SK129/CR04[13].

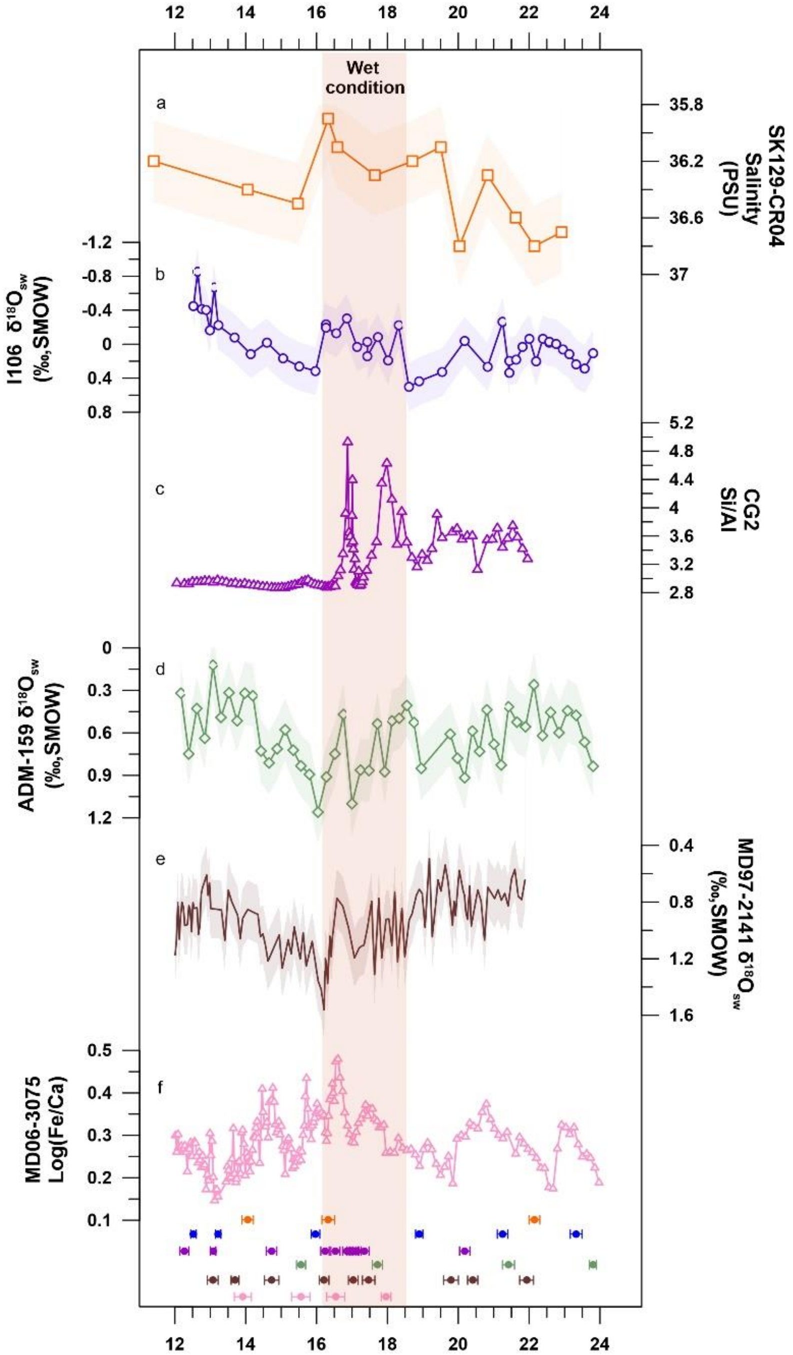

Supplementary Fig.4 The wet hydrological condition records in north of Equator from the Indo-Asian-Australian (IAA) monsoon region during the Heinrich Stadial (HS1). (a) Sea surface salinity (SSS) record from Core SK129-CR04 from the tropical Indian Ocean[13]. (b) $\delta^{18}O_{seawater}$ ($\delta^{18}O_{sw}$) records from Core I106 from the tropical Indian Ocean (this study). (c) Si/Al ratios from Core CG2 from the southern South China Sea (SCS) [15]. (d) $\delta^{18}O_{sw}$ records from Core ADM-9 from the southern Andaman Sea [11]. (e) $\delta^{18}O_{sw}$ records from Core MD97-2141 from the Sulu Sea[12]. (f) XRF-derived log (Fe/Ca) records from MD06-3075 at Mindanao[16].

Supplementary Table 1. Paleoclimatic records during the Early HS1 in the Indo-Asian-Australian monsoon region

No.	Core name	Latitude	Longitude	Proxy	Early HS1	References
1	SO93-126KL	19°58.40′N	90°02.03′E	Salinity	dry	Kudrass et al., 2001 [17]
2	BOB-24	15°34′N	87°9′E	$\delta^{18}O_{sw}$	dry	Liu et al., 2020 [18]
3	VM29-19	14.71°N	83.58°E	$\delta^{18}O_{sw}$	dry	Rashid et al., 2011 [19]
4	SK218/1	14°02′N	82°00′E	$\delta^{18}O_{sw}$	dry	Govil and Naidu, 2011 [20]
5	RC12-344	12.46°N	96.04°E	$\delta^{18}O_{sw}$	dry	Rashid et al., 2007 [21]
6	SO130-289KL	23°07.34′N	66°29.84′E	reflectance L*	dry	Deplazes et al., 2013 [22]
7	SK17	15°15′N	72°58′E	$\delta^{18}O_{sw}$	dry	Anand et al., 2008 [23]
8	SS-4018	13°12.80'N	53°15.40'E	biogenic silica flux	dry	Balaji et al., 2018 [24]
9	SK237-GC04	10°58.65′N	74°59.96′E	$\delta^{18}O_{sw}$	dry	Saraswat et al., 2013 [25]
10	GeoB12624-1	8°14'S	39°45'E	X-ray fluorescence, pollen	dry	Liu et al., 2018 [26]
11	GIK16160-3	18°14.47'S	37°52.11'E	$\delta^{18}O_{sw}$	dry	Wang et al., 2013 [27]
12	ADM-159	9.27°N	95.61°E	$\delta^{18}O_{sw}$	wet	Liu et al., 2021 [11]
13	SK129/CR04	6°29.65′N	75°58.68′E	$\delta^{18}O_{sw}$	wet	Mahesh and Banakar, 2014[13]
14	I106	6°14′N	90°00′E	$\delta^{18}O_{sw}$	wet	This study
15	SO189-119KL	3°31′N	96°19′E	$\delta^{18}O_{sw}$	dry	Mohtadi et al., 2014 [5]
16	SO189-144KL	1°09'N	98°04′E	$\delta^{18}O_{sw}$	dry	Mohtadi et al., 2014 [5]
17	SO189-39KL	0°47′S	99°54′E	$\delta^{18}O_{sw}$	dry	Mohtadi et al., 2014 [5]
18	GeoB 10042-1	7°06.81'S	104°38.58'E	$\delta^{18}O_{sw}$	dry	Mohtadi et al., 2010 [28]
19	Tiancai Lake	26°38′N	99°43′E	pollen	dry	Xiao et al. 2014 [29]
20	TCQH	25°07'N	98°34′E	Pollen	dry	Xiao et al. 2015 [30]
21	Sanbao Cave	31° 40'N	110° 26′E	Speleothems $\delta^{18}O$	dry	Chen et al., 2016 [31]
22	LJF	26° 37'N	110° 08'E	pollen	dry	Ma et al., 2022 [32]
23	SZY	26°46′N	119° 02′E	pollen	dry	Yue et al., 2012 [33]
24	Toushe	23°49'N	120°53'E	pollen, n-alkane-δD	dry	Li et al., 2013 [34]
25	MD05-2904	19°27′N	116° 15′E	pollen	dry	Chang et al., 2013 [35]
26	17940	20°07′N	117° 23′E	Salinity	dry	Wang et al., 1999 [36]
27	TGS-931	2°24.5′S	122°37'E	$\delta^{18}O_{sw}$	dry	Schröder et al., 2018 [37]
28	SO217-18519	0°34.3′S	118°6.9′E	$\delta^{18}O_{sw}$	dry	Schröder et al., 2018 [37]
29	SO217-18515	3°37.8'S	119°22'E	$\delta^{18}O_{sw}$	dry	Schröder et al., 2016 [38]
30	GeoB10069-3	9°36' S	120°55'E	$\delta^{18}O_{sw}$	dry	Gibbons et al., 2014 [39]
31	MD01-2378	13°4.95´ S	121°47´ E	$\delta^{18}O_{sw}$	dry	Schröder et al., 2016 [38]
32	MD98-2165	9°39´ S	118°20´ E	$\delta^{18}O_{sw}$	dry	Levi et al., 2007 [40]
33	Borneo SSC01	4°N	114°E	Speleothems $\delta^{18}O$	dry	Partin et al., 2007 [41]
34	VM33-80	7°51.7′S	123°E	^{232}Th flux, Particle flux	dry	Muller et al., 2010 [42]
35	Ball Gown Cave	17°20'S	125°E	Speleothems $\delta^{18}O$	dry	Denniston et al., 2013 [43]
36	Lake Challa	3°19'S	37°42'E	BIT index	dry	Verschuren et al., 2009 [44]
37	P178-15P	11° 57.3'N	44° 18' E	δDwax	dry	Tierney et al., 2013 [45]
38	Socotra	12° 31'N	54° E	Speleothems $\delta^{18}O$	dry	Shakun et al.,2007 [46]
39	Mawmlun	25° 15'N	91° 52' E	Speleothems $\delta^{18}O$	dry	Dutt et al., 2015 [47]
40	CG2	6.39°N	110.15°E	grain size, Si/Al, $\delta^{18}O_{sw}$	wet	Huang et al., 2019 [15]
41	MD97-2141	8.8 °N	121.3° E	$\delta^{18}O_{sw}$	wet	Rosenthal et al., 2003 [12]
42	MD06-3075	6°29'N	125°50'E	X-ray fluorescence	wet	Fraser et al., 2014 [16]
43	Lake Malawi	10 ° 01.1'S	34°11.2'E	X-ray fluorescence	dry	Castañeda et al., 2007 [48]

Supplementary Table 2. AMS ^{14}C ages and converted calendar ages for Core I106 in the tropical Indian Ocean.

No.	Depth/cm	Material	Measured ^{14}C age (BP)	Conventional ^{14}C age (BP)	Calendar calibrated age (cal BP)
1	5*	Mixed foraminifer	4160	4590±30	4705-4517
3	13	Mixed foraminifer	6510	6950±30	7335-7188
2	19	Mixed foraminifer	8320	8750±30	9322-9140
4	25*	Mixed foraminifer	10690	11130±40	12602-12443
5	31*	Mixed foraminifer	11460	11900±40	13296-13144
6	37	Mixed foraminifer	13470	13910±40	16088-15847
7	47	Mixed foraminifer	15970	16400±40	18998-18792
8	58*	Mixed foraminifer	17910	18350±50	21400-21098
9	69*	Mixed foraminifer	20050	20180±60	23488-23158
10	81	Mixed foraminifer	22400	22860±80	26319-26043
11	93*	Mixed foraminifer	24590	25030±90	28560-28236
12	101*	Mixed foraminifer	27820	28240±120	31562-31250
13	113	Mixed foraminifer	30130	30560±150	34377-34076
14	125*	Mixed foraminifer	31820	32250±200	36102-35648
15	133*	Mixed foraminifer	36370	36810±280	41028-40560
16	145	Mixed foraminifer	38870	39290±390	42429-42069
17	157*	Mixed foraminifer	42190	42620±560	44962-44092

*The AMS ^{14}C data have been published by Xu et al. (2022) [49].

Supplementary References

1. Gebregiorgis, D. et al. Southern Hemisphere forcing of South Asian monsoon precipitation over the past ~1 million years. *Nature Communications* **9**, 4702 (2018).
2. Singh, A., Jani, R.A. & Ramesh, R. Spatiotemporal variations of the δ18O–salinity relation in the northern Indian Ocean. *Deep Sea Research Part I: Oceanographic Research Papers* **57**, 1422-1431 (2010).
3. Delaygue, G., et al. Oxygen isotope/salinity relationship in the northern Indian Ocean. *Journal of Geophysical Research: Oceans* **106**, 4565-4574 (2001).
4. Achyuthan, H., et al. Stable isotopes and salinity in the surface waters of the Bay of Bengal: Implications for water dynamics and palaeoclimate. *Marine Chemistry* **149**, 51-62 (2013).
5. Mohtadi, M. et al. North Atlantic forcing of tropical Indian Ocean climate. *Nature* **509**, 76-80 (2014).
6. Yang, Y.P. et al. Meridional migration of Indian Ocean Monsoon precipitation during the early Holocene: Evidence from the Andaman Sea. *Quaternary Science Reviews* **267**, (2021).
7. Rashid, H., Flower, B.P., Poore, R.Z. & Quinn, T.M. A ~25ka Indian Ocean monsoon variability record from the Andaman Sea. *Quaternary Science Reviews* **26**, 2586-2597 (2007).
8. Bolton, C.T. et al. A 500,000 year record of Indian summer monsoon dynamics recorded by eastern equatorial Indian Ocean upper water-column structure. *Quaternary Science Reviews* **77**, 167-180 (2013).
9. Bemis, B.E., Spero, H.J., Bigma, J. & Lea, D.W. Reevaluation of the oxygen isotopic composition of planktonic foraminifera: Experimental results and revised paleotemperature equations. *Paleoceanography* **13**, 150-160 (1998).
10. Thirumalai, K., Quinn, T. M., & Marino, G. Constraining past seawater $\delta^{18}O$ and temperature records developed from foraminiferal geochemistry. Paleoceanography 31, 1409-1422 (2016).
11. Liu, S. et al. Paleoclimatic responses in the tropical Indian Ocean to regional monsoon and global climate change over the last 42 kyr. *Marine Geology* **438**, 106542 (2021).
12. Rosenthal, Y., Oppo, D.W. & Linsley, B.K. The amplitude and phasing of climate change during the last deglaciation in the Sulu Sea, western equatorial Pacific. *Geophysical Research Letters* **30**, L024070 (2003).
13. Mahesh, B.S. & Banakar, V.K. Change in the intensity of low-salinity water inflow from the Bay of Bengal into the Eastern Arabian Sea from the Last Glacial Maximum to the Holocene: Implications for monsoon variations. *Palaeogeography, Palaeoclimatology, Palaeoecology* **397**, 31-37 (2014).
14. Epstein, S., Buchsbaum, R.M., Lowenstam, H.A. & Urey, H.C. Revised carbonate-water isotopic temperature scale. *Bulletin of the Geological Society of America* **64**, 1315-1325 (1953).
15. Huang, J., Wan, S.M., Li, A.C. & Li, T.G. Two-phase structure of tropical hydroclimate during Heinrich Stadial 1 and its global implications. *Quaternary Science Reviews* **222**, 105900 (2019).
16. Fraser, N. et al. Precipitation variability within the West Pacific Warm Pool over the past 120ka: Evidence from the Davao Gulf, southern Philippines. *Paleoceanography* **29**, PA002599 (2014).
17. Kudrass, H.R. et al. Modulation and amplification of climatic changes in the Northern Hemisphere by the Indian summer monsoon during the past 80 k.y. *Geology* **29**, 63-66 (2001).
18. Liu, S.F. et al. Reconstruction of monsoon evolution in southernmost Sumatra over the past 35 kyr and its response to northern hemisphere climate changes. Progress in Earth and Planetary Science 7, 1-13 (2020).
19. Rashid, H., England, E., Thompson, L. & Polyak, L. Late Glacial to Holocene Indian Summer Monsoon Variability Based upon Sediment Records Taken from the Bay of Bengal. *Terrestrial, Atmospheric and Oceanic Sciences* **22**, 215-228 (2011).
20. Govil, P. & Naidu, P.D. Variations of Indian monsoon precipitation during the last 32kyr reflected in the surface hydrography of the Western Bay of Bengal. *Quaternary Science Reviews* **30**, 3871-3879 (2011).
21. Rashid, H., Flower, B.P., Poore, R.Z. & Quinn, T.M. A ~25ka Indian Ocean monsoon variability record from

the Andaman Sea. *Quaternary Science Reviews* **26**, 2586-2597 (2007).

22. Deplazes, G. et al. Links between tropical rainfall and North Atlantic climate during the last glacial period. *Nature Geoscience* **6**, 213-217 (2013).
23. Anand, P. et al. Coupled sea surface temperature-seawater $\delta^{18}O$ reconstructions in the Arabian Sea at the millennial scale for the last 35 ka. *Paleoceanography* **23**, PA4207 (2008).
24. Balaji, D., Bhushan, R. & Chamyal, L.S. Variations of the Somali upwelling since 18.5 ka BP and its relationship with southwest monsoon rainfall. *Climate of the Past* **14**, 1331-1343 (2018).
25. Saraswat, R. et al. A first look at past sea surface temperatures in the equatorial Indian Ocean from Mg/Ca in foraminifera. *Geophysical Research Letters* **32**, L24605 (2005).
26. Liu, X.T., Rendle-Bühring, R. & Henrich, R. High-and low-latitude forcing of the East African climate since the LGM: Inferred from the elemental composition of marine sediments off Tanzania. *Quaternary Science Reviews* **196**, 124-136 (2018).
27. Wang, Y.M.V. et al. Northern and southern hemisphere controls on seasonal sea surface temperatures in the Indian Ocean during the last deglaciation. *Paleoceanography* **28**, 619-632 (2013).
28. Mohtadi, M. et al. Glacial to Holocene surface hydrography of the tropical eastern Indian Ocean. *Earth and Planetary Science Letters* **292**, 89-97 (2010).
29. Xiao, X.Y. et al. Latest Pleistocene and Holocene vegetation and climate history inferred from an alpine lacustrine record, northwestern Yunnan Province, southwest China. *Quaternary Science Reviews* **86**, 35-48 (2018).
30. Xiao, X.Y. et al. Vegetation, fire, and climate history during the last 18 500 cal a BP in south-western Yunnan Province, China. *Journal of Quaternary Science* **30**, 859-869 (2015).
31. Cheng, H. et al. The Asian monsoon over the past 640,000 years and ice age terminations. *Nature* **534**, 640-646 (2016).
32. Ma, T. et al. Intensified climate drying and cooling during the last glacial culmination (20.8-17.5 cal ka BP) in the south-eastern Asian monsoon domain inferred from a high-resolution pollen record. *Quaternary Science Reviews* **278**, 107371(2022).
33. Yue, Y.F. et al. A continuous record of vegetation and climate change over the past 50,000 years in the Fujian Province of eastern subtropical China. *Palaeogeography, Palaeoclimatology, Palaeoecology* **365-366**, 115-123 (2012).
34. Li, H.C. et al. Paleoclimate variability in central Taiwan during the past 30Kyrs reflected by pollen, $\delta^{13}C$,TOC, and n-alkane-δD records in a peat sequence from Toushe Basin. *Journal of Asian Earth Sciences* **69**, 166-176 (2013).
35. Chang, L., Luo, Y. & Sun, X. Paleoenvironmental change base on a pollen record from deep sea core MD05-2904 from the northern South China Sea during the past 20000 years. *Chinese Science Bulletin* **58**, 3079-3087 (2013).
36. Wang, L.J. et al. East Asian monsoon climate during the Late Pleistocene: high-resolution sediment records from the South China Sea. *Marine Geology* **156**, 245-284 (1999)
37. Schröder, J.F. et al. Deglacial Warming and Hydroclimate Variability in the Central Indonesian Archipelago. *Paleoceanography and Paleoclimatology* **33**, 974-993 (2018).
38. Schröder, J.F., Holbourn, A., Kuhnt, W. & Küssner, K. Variations in sea surface hydrology in the southern Makassar Strait over the past 26 kyr. *Quaternary Science Reviews* **154**, 143-156 (2016).
39. Gibbons, F.T. et al. Deglacial $\delta^{18}O$ and hydrologic variability in the tropical Pacific and Indian Oceans. *Earth and Planetary Science Letters* **387**, 240-251 (2014).
40. Levi, C. et al. Low-latitude hydrological cycle and rapid climate changes during the last deglaciation. *Geochemistry, Geophysics, Geosystems* **8**, Q05N12 (2007).

41. Partin, J.W. et al. Millennial-scale trends in west Pacific warm pool hydrology since the Last Glacial Maximum. *Nature* **449**, 452-457 (2007)
42. Muller, J., McManus, J.F., Oppo, D.W. & Francois R. Strengthening of the Northeast Monsoon over the Flores Sea, Indonesia, at the time of Heinrich event 1. *Geology* **40**, 635-638 (2012).
43. Denniston, R.F. et al. North Atlantic forcing of millennial-scale Indo-Australian monsoon dynamics during the Last Glacial period. *Quaternary Science Reviews* **72**, 159-168 (2013).
44. Verschuren, D. et al. Half-precessional dynamics of monsoon rainfall near the East African Equator. *Nature* **462**, 637-642 (2009)
45. Tierney, J.E., Pausata, F.S.R. & deMenocal, P. Deglacial Indian monsoon failure and North Atlantic stadials linked by Indian Ocean surface cooling. *Nature Geoscience* **9**, 46-50 (2015).
46. Shakun, J.D. et al. A high-resolution, absolute-dated deglacial speleothem record of Indian Ocean climate from Socotra Island, Yemen. *Earth and Planetary Science Letters* **259**, 442-456 (2007)
47. Dutt, S. et al. Abrupt changes in Indian summer monsoon strength during 33800 to 5500 years B.P. *Geophy. Res. Lett.* **42**, 5526-5532 (2015).
48. Castaeda, I.S. , Werne, J.P. & Johnson, T.C. Wet and arid phases in the southeast african tropics since the last glacial maximum. *Geology* **35**, 823-826 (2007).
49. Xu, X.D. et al. An Intertropical Convergence Zone shift controlled the terrenstrial material supply on the Ninetyeast Ridge, *Clim. Past.* **18**, 1369-1384 (2022).
