# Supplementary material for: A contracting Intertropical Convergence Zone during the Early Heinrich Stadial 1
Source: Nat Commun. 2023 Aug 4;14:4695. doi: 10.1038/s41467-023-40377-9 (PMC10403598; doi:10.1038/s41467-023-40377-9)
Supplement: Supplementary file 3 — Description of Additional Supplementary Files Document [file 41467_2023_40377_MOESM3_ESM.pdf]

Description of Additional Supplementary Files Document

Supplementary Dataset 1 - modern 18Oseawater and sea surface salinity (SSS) values from the tropical Indian Ocean.

Supplementary Dataset 2 - Age models for all cores.
